# Treadmill or Overground? Cognitive–Motor Interference Effects in Healthy Older Individuals

**DOI:** 10.1111/ejn.70724

**Published:** 2026-10-25

**Authors:** Lara J. Papin, Julius Welzel, Jessica Koschate‐Storm, Tania Zieschang, Karsten Witt, Stefan Debener

**Affiliations:** ^1^ Department of Psychology, Neuropsychology Lab Carl von Ossietzky University Oldenburg Oldenburg Germany; ^2^ Department of Neurology University Hospital Schleswig‐Holstein and Kiel University Kiel Germany; ^3^ Department for Health Services Research, Geriatric Medicine, School of Medicine and Health Sciences Carl von Ossietzky University Oldenburg Oldenburg Germany; ^4^ Cluster Excellence Hearing4all Carl von Ossietzky University Oldenburg Oldenburg Germany; ^5^ Department of Neurology Carl von Ossietzky University Oldenburg Oldenburg Germany; ^6^ University Clinic for Neurology, at the Evangelical Hospital Oldenburg Germany; ^7^ Center for Neurosensory Sciences and Systems Carl von Ossietzky University Oldenburg Oldenburg Germany; ^8^ Fraunhofer Institute Digital Media Technology Branch Hearing Speech & Audio Technology Oldenburg Germany

**Keywords:** cognitive motor interference, cognitive resource theory, gait, mobile EEG, treadmill

## Abstract

Walking is increasingly understood as a cognitively demanding activity requiring the integration of sensory processing, motor control and executive functions. When combined with a concurrent cognitive task (dual‐tasking), cognitive–motor interactions may lead to changes in gait, behaviour and neurophysiology. In healthy aging, reduced motor automaticity may result in greater involvement of top–down control, increasing susceptibility to cognitive–motor interference and fall risk, even in the absence of clinical impairment. To explore real‐world demands, we compared dual‐tasking during outdoor overground walking and during indoor treadmill walking, using behavioural assessment of cognitive task performance, mobile electroencephalography and motion tracking with wearable sensors. Thirty‐six older adults performed an auditory Go/No‐Go task during both walking conditions and in a seated control condition. Compared to a stationary control condition, results confirmed significant dual‐task interference effects during both walking conditions, and this effect was more pronounced during overground walking. The results suggest that overground walking imposes greater demands, leading to increased competition between cognitive and motor processes for shared cognitive resources. The consideration of natural conditions helps to identify the cognitive demands of locomotion.

## Graphical abstract

Although treadmill walking is widely used to study cognitive–motor interactions, its reflection of natural gait remains unclear. We compared dual‐tasking during treadmill and natural gait using mobile EEG and an auditory Go/No‐Go task. Natural gait imposed greater demands, increasing competition between cognitive and motor processes for shared cognitive resources.

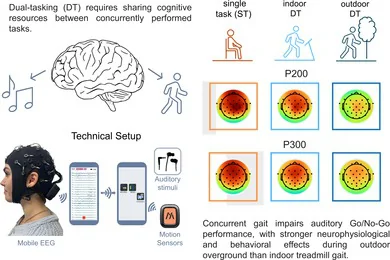

**Abbreviations**
CMI: Cognitive–motor interference
DT: Dual‐tasking
EEG: Electroencephalography
ERP: Event‐related potential
IMU: Inertial measurement unit
MRT: Multiple resource theory
SD: Standard deviation
ST: Single‐tasking

## 1 Introduction

Everyday walking often occurs alongside additional tasks like conversing, navigating or monitoring the environment. Although gait is frequently considered automatic, it relies on ongoing interactions between cognitive, sensory and motor systems (Bayot et al. 2018; Liu et al. 2023; Yogev‐Seligmann et al. 2008). Consequently, walking and concurrent cognitive tasks may interfere and compete for shared cognitive resources (Al‐Yahya et al. 2011).

Dual‐tasking (DT) is commonly defined as the simultaneous execution of two tasks that can be executed and assessed independently (McIsaac et al. 2015). In cognitive–motor DT contexts, such as walking while talking, performance depends on the demands of both tasks (Al‐Yahya et al. 2011; Plummer et al. 2015; Plummer and Eskes 2015). Compared with single‐task (ST) conditions, DT performance may remain stable, improve or decline. Performance improvement or facilitation effects have been linked to increased arousal and more efficient resource allocation during moderate physical activity (Atakanova et al. 2023; Patelaki, Foxe, Mantel, et al. 2023), whereas performance decline is interpreted as cognitive–motor interference (CMI) resulting from limited processing capacity and competition for shared resources (Al‐Yahya et al. 2011; Plummer et al. 2013). According to Wickens' multiple resource theory (MRT), interference occurs when concurrent tasks compete for overlapping cognitive resources across different dimensions, such as processing stage (e.g., perception), sensory modality (e.g., visual) and processing code (e.g., spatial) (Wickens 2008, 2024). However, the MRT is not specifically developed to consider cognitive–motor interactions during gait. An extension of MRT aims to better capture the demands of walking. Here, DT gait requires somatosensory and vestibular sensory modalities and a framework for quantifying task demand and conflict is provided (Cinar et al. 2022). The extent of interference during DT depends on both, task demands and the degree to which tasks compete for shared resources. During walking, such competition may be particularly relevant when locomotion requires visuospatial monitoring, environmental adaptation or increased motor control.

Gait parameters such as speed and variability are sensitive to concurrent cognitive load (Arpaia et al. 2024; Hausdorff et al. 2008; Hobert et al. 2017; Lövdén et al. 2008; Simoni et al. 2013). Cognitive performance may also decline under DT conditions, as indicated by slower response times (Hazamy et al. 2017; Papin et al. 2026; Protzak et al. 2021) and reduced task accuracy (P.‐C. Kao and Pierro 2021; Koren et al. 2022; Liston et al. 2014; Patel et al. 2014; Penati et al. 2020). Complementing these behavioural findings, electroencephalography (EEG) based gait studies show consistent DT effects on event‐related potentials (ERP). The effects manifest as reduced P3 (e.g., (De Vos et al. 2014; Debener et al. 2012; Jacobsen et al. 2025; Reiser et al. 2019) and P2 amplitudes (Reiser et al. 2021) during DT compared to ST. These ERP amplitude reductions are interpreted as reflecting the allocation of limited cognitive resources to the execution of the motor task (Nenna et al. 2021; Rosenfeld et al. 1993). In addition, higher motor demands may further disrupt stimulus updating processes (Reiser et al. 2021), which have been linked to P2 and P3 ERP components (Kok 2001; Polich 2007). Overall, these findings indicate that DT walking taxes shared cognitive resources to their capacity limits, leading to measurable changes in behaviour, gait and neural processing, depending on task demands and characteristics.

In contrast to the common finding of cognitive‐locomotion interference, a recent study reported that treadmill walking enhanced visual cognitive processing (2023 Patelaki, Foxe, Mantel, et al. 2023). This facilitation effect may reflect an arousal‐related optimisation of cognitive resource allocation. However, according to the framework mentioned above (Cinar et al. 2022), overground walking requires greater use of visuospatial and vestibular resources; thus, it may lead to CMI when combined with concurrent cognitive tasks by placing greater demands on shared resources than treadmill walking. Moreover, treadmill and overground gait differ fundamentally in gait dynamics (Carpinella et al. 2010; Hollman et al. 2016). Treadmill walking imposes external pacing (Simoni et al. 2013), resulting in more stable and less variable gait patterns, whereas overground walking exhibits greater variability (Hollman et al. 2016). It is likely that treadmill walking does not fully simulate natural locomotion demands, yet most DT gait studies rely on treadmill‐based paradigms (Gramann et al. 2010; Maidan et al. 2019; Malcolm et al. 2015). While some studies have examined overground DT (De Vos et al. 2014; Debener et al. 2012; Reiser et al. 2021; Jacobsen et al. 2025), direct comparisons between DT treadmill and natural overground walking are rare.

Few studies evaluated differential DT effects between treadmill and indoor overground walking (Wrightson et al. 2020; Wrightson and Smeeton 2017), and only one study has investigated these effects during outdoor overground walking (Papin et al. 2026). These studies were exclusively conducted with young adults. In contrast, older adults typically show greater DT performance decrements (Hausdorff et al. 2008; Montero‐Odasso et al. 2012), which may reflect age‐related cognitive decline (Beurskens and Bock 2012) and reduced neural processing capacity (Rosso et al. 2025). Moreover, none of the studies directly comparing DT treadmill and overground walking included EEG measures, leaving unclear how neural correlates of cognition are modulated across these walking conditions.

The present study investigates DT walking across treadmill and overground conditions in older adults. We examine how motor and environmental context influence CMI by directly comparing indoor treadmill walking (DT Indoor), natural outdoor walking (DT Outdoor) and a stationary sitting condition (ST) (see Figure 2). Participants perform an auditory Go/No‐Go task under ST and DT conditions while neural (mobile EEG), behavioural and gait measures are recorded. We evaluated the following hypotheses:

1. If gait dynamics differ between locomotion contexts, then gait speed and variability are expected to differ between DT Indoor and DT Outdoor.
2. If concurrent gait affects cognitive performance, then response times will increase and Go/No‐Go sensitivity (d‐prime) will decrease from ST to DT. If treadmill gait imposes lower cognitive demands than overground gait, then response times will increase and d‐prime will decrease from DT Indoor to DT Outdoor.
3. If concurrent gait modulates neural processing of the auditory task, P2 and P3 ERP amplitudes are smaller in DT versus ST conditions. If treadmill gait affects this neural processing less strongly than overground gait, then P2 and P3 amplitudes will decrease from DT Indoor to DT Outdoor.

## 2 Material and Methods

### 2.1 Participants

Thirty‐six healthy older adults participated in the study (66.6 ± 7.2 years; 20 female, 16 male; 28 right‐handed, 3 left‐handed, 5 ambidextrous; 17.5 ± 4.2 years of education). All participants provided written informed consent and received an honorarium of €12 per hour. Due to technical errors and unexpected weather changes resulting in data loss, the data of six participants had to be excluded; their EEG and/or motion data were incomplete. The final sample therefore consisted of *N* = 30 participants (67.0 ± 7.3 years; range 56–85 years; 17 female, 13 male; 23 right‐handed, 3 left‐handed, 4 ambidextrous; 17.6 ± 4.4 years of education). All participants reported normal or corrected‐to‐normal vision and normal hearing. Individuals with uncorrected visual impairments, hearing difficulties, neurological or psychiatric diagnoses, or medication use affecting cognitive or motor function were excluded. Screening tests for physical and psychological health revealed no signs of cognitive impairment (Montreal Cognitive Assessment [MoCA], score ≥ 25), depression (Beck's Depression Inventory‐II [BDI‐II], score < 14), impaired gait function (Functional Gait Assessment [FGA], score ≥ 22) or impaired hand function (Jebsen–Taylor Hand Function Test [JHFT], cutoff > 38 s). Mental and physical health, which was assessed using the Short Form Health Survey (SF‐12), revealed no clinically relevant impairments indicated in the sample. Additionally, none of the participants reported gait or balance impairments. Physical activity levels and treadmill experience were assessed using a questionnaire. The majority of participants reported being physically active more than four times per week (*n* = 10) or two to three times per week (*n* = 12), whereas a smaller proportion reported being active once or less per week (*n* = 8). Treadmill experience was generally limited: Most participants reported little (*n* = 12) or no (*n* = 9) prior experience, less reported moderate experience (*n* = 9) and none reported regular treadmill use. Furthermore, all participants indicated that they had slept at least 5 hours the night before the experiment and reported no alcohol or drug use on the day of testing or the preceding day. The experimental procedure was approved by the Medical Ethics Committee of Carl von Ossietzky University Oldenburg (Nr. 2023‐156).

### 2.2 Materials

Two smartphones were used for data acquisition. One phone (Samsung Galaxy S21 5G, Software: Android version 13.0.0) was used to execute the experiment (using Presentation version: 23.0, build 10.27.21 computer, version: 3.0.6. smartphone; Neurobehavioral Systems Inc., Berkeley, California, RRID: 143 SCR_002521) and stream sensor signals into the local area network, using the SENDA app (version 1.2.; Haupt et al. 2026). The second phone (Samsung Galaxy S24+, Software: Android version 14.0.1) was used to collect EEG signals (using SmartingProApp version 3.3.0, mBrainTrain, Belgrade, Serbia) and write all time‐synchronised sensor streams into a single xdf file, using the RECORDA app (version 1.0.00.9; Haupt et al. 2026; see Figure 1). Participants held one of the smartphones in their hands throughout the experiment and used the device as a response button. They were asked not to look to the screen and to keep their gaze directed ahead along the walking path. The other smartphone was held by the experimenter. DT Indoor walking was performed on an electronic treadmill (M‐Gait treadmill; Motek Medical B.V., Amsterdam, the Netherlands, software: D‐FLOW, version 36.2). EEG data were recorded using a wireless amplifier (SmartingPro, mBrainTrain, Belgrade, Serbia) mounted to the participants' head capturing 32‐channel EEG data using passive wet electrodes (Ag/AgCl sintered) placed at 10–20 locations F3, F4, C3, C4, P3, P4, O1, O2, F7, F8, T7, T8, P7, P8, Fz, Cz, Pz, POz, FP1, FP2, FC1, FC2, CP1, CP2, FC5, FC6, CP5, CP6, FT9, FT10, TP9, TP10. Reference and ground electrodes were placed at positions PCz and AFz, respectively. Sampling rate was set to 500 Hz. Easycap electrode gel (Abralyt HiCl) as well as light rubbing with a cotton swab were used to lower impedances of all electrodes until a good conductivity (< 10 kΩ) was achieved. To record motion data, nine‐axis inertial measurement unit (IMU) sensors (Movella, Xsens DOT, Hardware: v2.0; Firmware 3.0.0) were placed on both feet using medical tape (see Figure 2).

**FIGURE 1:**
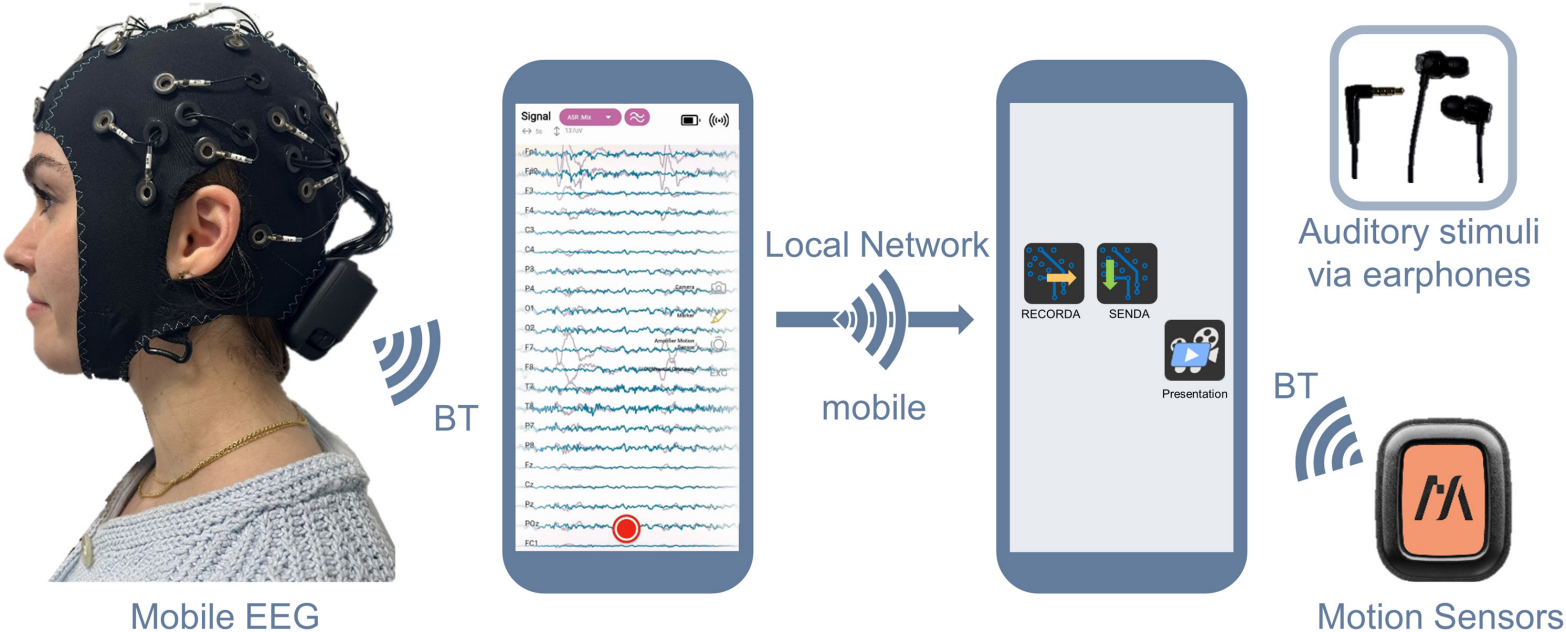
Technical setup: Data from a 32‐channel EEG cap were transmitted via Bluetooth to Smartphone 1 and recorded using the Smarting Pro app, then streamed over a local Wi‐Fi network to Smartphone 2. IMU data were sent via Bluetooth directly to Smartphone 2. The auditory Go/No‐Go task (presented using the NBS Presentation app) ran on Smartphone 2, where all signals were recorded and time‐synchronised using the RECORDA app.

**FIGURE 2:**
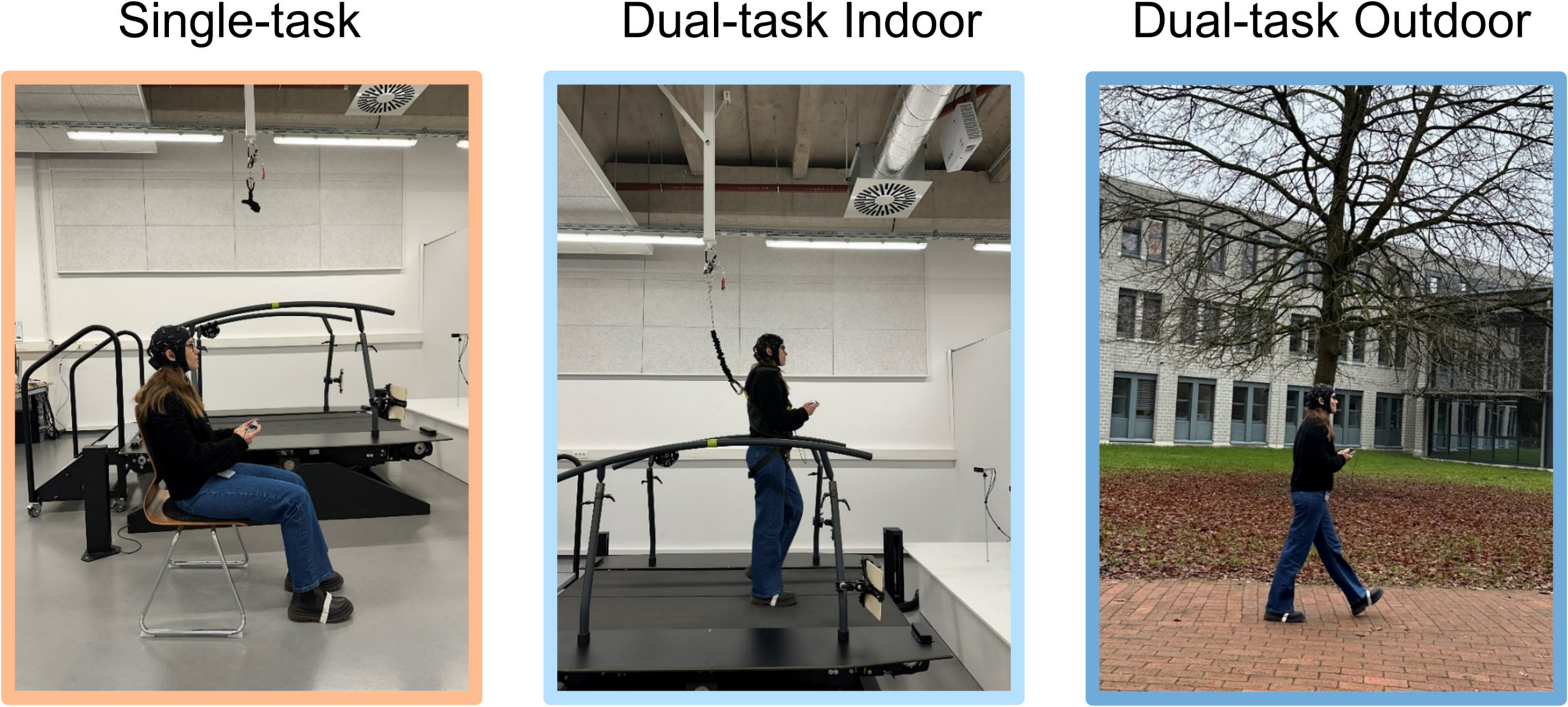
Schematic overview of the three experimental conditions: Dual‐task (DT) Outdoor, Dual‐task (DT) Indoor and Single‐task (DT). Each condition was divided into two runs of 5 min (10 min total per condition).

Test of Attentional Performance—Mobility (TAP‐M) version 1.3.2 was used on a Laptop (Toshiba Intel R Core i5 CPU M 520 at 2.40GHz; software: Windows 10 22H2, system build: 19045.3996), in a quiet room without distractions. Four subtests were utilised: Executive Control, Divided Attention (visual), Go/No‐Go (visual) and Distractibility. To register responses, either one or both of the external response buttons (5 cm × 5 cm) provided by the TAP materials were pressed.

### 2.3 Tasks and Procedure

Data were collected both outdoors on the University campus and indoors in a laboratory on campus equipped with the treadmill. Experiments were not conducted during heavy rain or storm. Outside temperatures ranged between 4 °C and 26 °C (M = 14.8 ± 5.8). Participants were invited twice; the first session took place in the laboratory; the second session took place in both the laboratory and outdoors on the University campus (see Figure S1).

During the first session a screening for physical and psychological health was conducted (MoCA, BDI‐II, FGA, JHFT, SF‐12, TAP‐M). Data collected during this session will be used in another project (RTG 2783 Neuromodulation). During the second session EEG, IMU and Go/No‐Go data were collected. Before data collection, participants completed a questionnaire gathering demographic information, prior experience with treadmill walking and weekly exercise habits (see Section 2.1). Following this questionnaire, participants underwent a gait speed assessment where they were asked to walk 8 m three times. The time they took to walk the 8 m was recorded to determine comfortable overground walking speed. Afterwards, they were introduced to the treadmill and completed a 3‐min familiarisation period. During the familiarisation period, they were instructed to select a comfortable walking speed (M = 3.80 ± 0.77 km/h) which they maintained throughout the experiment. Then, an auditory practice Go/No‐Go trial was played to the participants binaurally through in‐ear headphones (Sennheiser CX 300S). Volume was individually adjusted to participants' preferred loudness and kept constant for the duration of the experiment. No specific control for ambient environmental noise was implemented in neither the indoor nor outdoor condition. This choice ensured that experimental conditions closely resembled real‐world walking situations. Importantly, most participants reported that surrounding environmental sounds were barely noticed, due to the use of in‐ear headphones throughout the experiment. Following the previously described familiarisation period, participants performed the Go/No‐Go task under three conditions: sitting (ST; A), walking on the treadmill indoors (DT Indoor; B) and walking outdoors (DT Outdoor; C) (see Figure 2). Each condition lasted 5 min and was performed twice, resulting in six consecutive runs per participant. The runs were arranged such that the three conditions were presented once in a given order and then repeated in reverse order (e.g., A‐B‐C‐C‐B‐A) to compensate within participant order effects. Condition order was counterbalanced across participants using six predefined sequences (ABCCBA, ACBBCA, BACCAB, BCAACB, CABBAC, CBAABC). Breaks between runs were self‐timed. Brakes were typically longer when transitioning to or from the DT Outdoor (C). This was due to the fact that DT Outdoor was conducted on an outdoors walking path (see Figure S1), whereas ST (A) and DT Indoor (B) took place in a laboratory. The outdoor walking path was located directly next to the laboratory building, accessible via a short elevator ride and a brief walking distance of less than 100 m.

The auditory Go/No‐Go task consisted of three pure tones (800 Hz, 1150 Hz and 1500 Hz), each lasting 80 ms, with inter‐stimulus intervals varying between 1000 and 1500 ms (uniform distribution with 125 ms distance). Participants were instructed to tap the smartphone screen whenever the current tone matched the previous one (Go) and to withhold response if the tone differed (No‐Go). No‐Go tones occurred with a probability of 15%, while Go tones occurred with an 85% probability. Participants were asked to keep their gaze centred at eye level throughout the tasks. Those with corrected vision wore their glasses.

After completing both runs of each condition, participants filled out a short version of the NASA‐TLX (National Aeronautics and Space Administration—Task Load Index) separately for each condition (Hart and Staveland 1988). This questionnaire assessed subjective task difficulty by measuring perceived cognitive and physical demands. It was used to evaluate whether the experienced mental load varied across conditions. To assess performance on the auditory task, we calculated the number of hits, misses, correct rejections, false alarms, d‐prime and response times following correct trials (hits) for each participant and condition respectively; exact definitions and calculation are provided in the following section (Section 2.4).

### 2.4 Data Analysis

Data analysis was performed offline using MATLAB (1984–2020 The MathWorks Inc. Natick, Massachusetts, United States, Version R2025a 9.9.0, RRID: SCR_001622). and the MATLAB toolbox EEGLAB (version 2025.0.0, RRID:SCR_007292) (Delorme and Makeig 2004). BIDS (RRID:SCR:016124) (Gorgolewski et al. 2016) extended for EEG data (Pernet et al. 2019) and motion data (Jeung et al. 2024) was used to organise the data recorded in the current study.

Auditory Go/No‐Go task data, including stimulus type, participant responses and corresponding timestamps, were analysed by first labelling each trial as either a Go or No‐Go trial. A Go trial followed by a response was classified as a *hit*; if no response occurred, it was classified as a *miss*. A No‐Go trial followed by a response was labelled a *false alarm*, while one without a response was labelled a *correct rejection*. Response times were calculated for all hits by taking the latency difference between the stimulus onset and the participant's response. Only response times between 200 ms and 900 ms were considered valid. As a measure of sensitivity, d‐prime scores were calculated for each condition individually. This was done by computing the inverse normal distribution (z) of the hit rate (z(H)) and the false alarm rate (z(F)) for each participant and condition individually and then subtracting z(F) from z(H): *d‐prime = z*(*H*) − *z*(*F*). Higher d‐prime values thus indicate more accurate auditory task performance. In addition, response bias was estimated using the centred response criterion (c). This was calculated by adding *z*(*F*) and *z*(*H*) and multiplying the result by −½: *c =* −½ × (*z*(*H*) + *z*(*F*)).

After raw data import the channel location coordinates were added. Channels that had abnormally high joint probability (threshold = 3 standard deviation [SD]), kurtosis (threshold = 3 SD) or variance (larger than mean +3 SD compared to the mean of all channels) and flatline channels were rejected. On average, 1.03 ± 0.67 (min: 0, max: 2) channels were rejected per participant, and after completion of preprocessing, replaced by spherical interpolation. Afterwards, data were re‐referenced to TP9 and TP10. Thereafter a 1 Hz zero‐phase, non‐causal high‐pass filter (finite impulse response [FIR] filter, high‐pass filter, transition bandwidth: 1 Hz, passband edge: 1 Hz, cutoff frequency: 0.5 Hz, order: 827) followed by a 30 Hz zero‐phase, non‐causal low‐pass filter (FIR filter, low‐pass filter, transition bandwidth: 7.5 Hz, passband edge: 30 Hz, cutoff frequency: 33.75 Hz, order: 111) were applied to the data. Then data were decomposed using independent component analysis (ICA, pop_runica, max. 512 steps, extended InfoMax algorithm). ICA weights were applied to the unfiltered, continuous dataset. ICLabel (Pion‐Tonachini et al. 2019) was used to identify components representing artefacts. Components were removed if their probability of representing artefactual activity (Muscle, Eye, Heart) was above 80%. As a result, 5.35 ± 2.37 (min: 1, max: 9) components were removed (eye: 2.03 ± 0.31, muscle: 3.29 ± 2.28, heart: 0.03 ± 0.18). Subsequently, a 0.3 Hz zero‐phase, non‐causal high‐pass (FIR filter, high‐pass filter, transition bandwidth: 0.3 Hz, passband edge: 0.3 Hz, cutoff frequency: 0.15 Hz, order: 2751) followed by a 30 Hz zero‐phase, non‐causal low‐pass filter (FIR filter, point low‐pass filter, transition bandwidth: 7.5 Hz, passband edge: 30 Hz, cutoff frequency: 33.75 Hz, order: 111) were applied to the data. Epochs ranging from −200 to 800 ms around tone onset were extracted and baseline corrected (−200 to 0 ms). ERPs were calculated from extracted epochs around stimulus onset. This was done for each condition (ST/DT Indoor/DT Outdoor) and stimulus type (Go/No‐Go) individually, only including correctly labelled responses (hit/correct rejection). ERP amplitudes at channel Cz were determined for each participant and condition by finding the maximum deviation from zero between 150 and 300 ms for P2, between 300 and 600 ms for P3, and the minimum deviation from zero between 50 and 200 ms for N1. To calculate the P2, P3 and N1 amplitude, a time window for each participant and condition was chosen (peak maximum (for P2/P3) ± 25 ms or peak minimum (for N1) ± 25 ms), respective values were calculated by averaging across this time window.

Accelerometer data were processed as follows. The first and last 15 s of each experimental block were discarded to remove transient effects. The remaining signal was filtered using a 0.5–3 Hz zero‐phase, non‐causal band‐pass filter (2nd‐order Butterworth infinite impulse response [IIR] filter; lower cutoff frequency: 0.5 Hz; upper cutoff frequency: 3 Hz). Stride events were identified via peak detection. Valid peaks were defined by a temporal separation of minimum 0.6 s and maximum 2 s from their nearest neighbour, and an amplitude exceeding a threshold set at 10% of the maximum signal magnitude (computed from the filtered accelerometer data). Stride intervals were calculated between consecutive peaks, and stride‐time variability was quantified as their SD. For Figure 3C, stride times were normalised for each participant by dividing individual stride times by their respective mean stride time. The resulting values were centred around 1 s and then averaged across participants. All analyses were conducted separately for each participant and locomotion condition using non‐normalised data. Visual inspection confirmed the validity of the step detection procedure.

**FIGURE 3:**
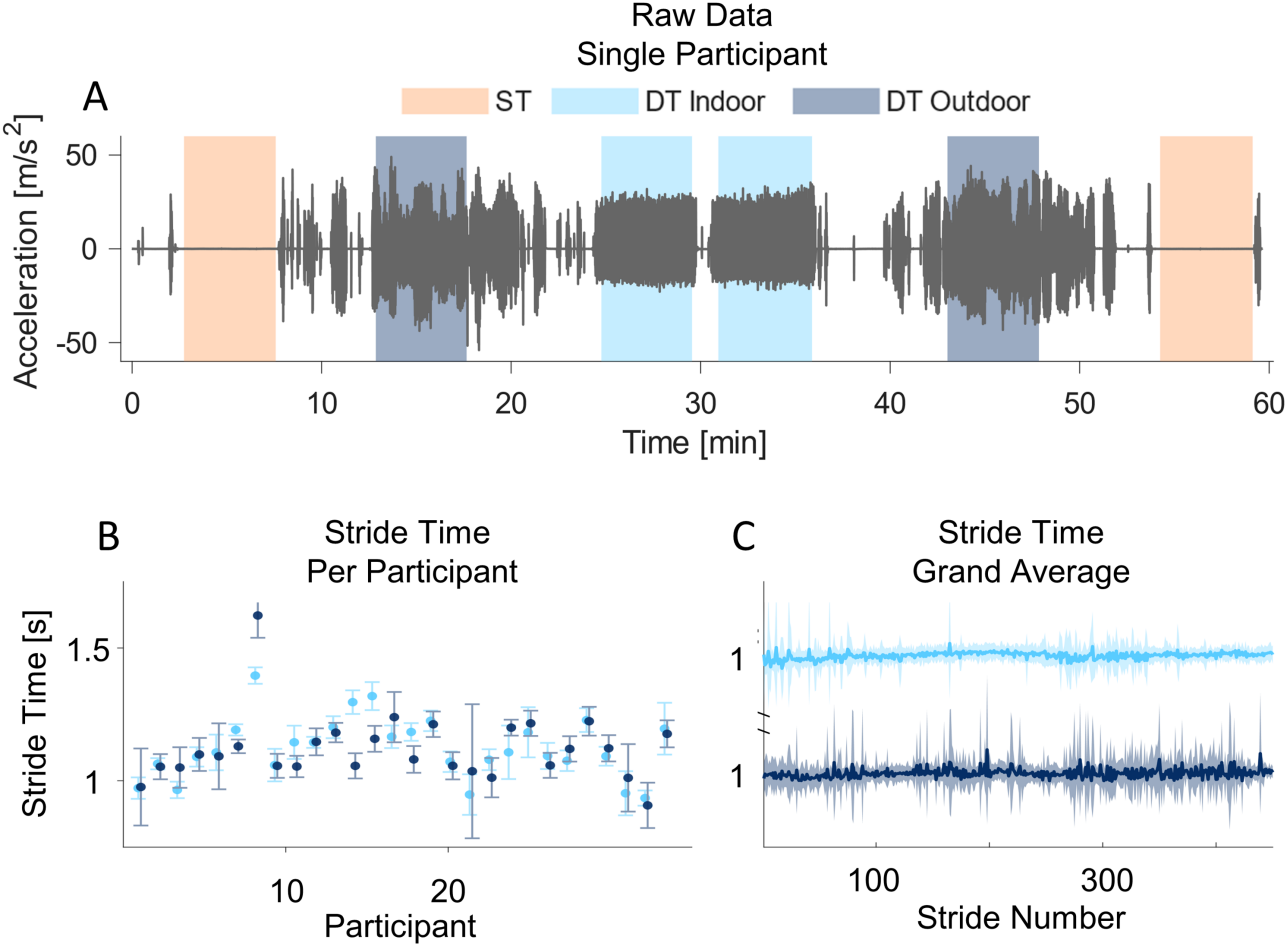
A provides an overview of the movement data recorded from a single participant over the whole experiment, and the stride‐time measures that were derived for all participants (Figure 3B, C). As can be seen, the different experimental conditions resulted in strong variations in linear acceleration. Figure 3A shows an exemplary display of one accelerometer axis of one participant across the whole experimental procedure. The boxes mark the time during which a specific condition took place (orange: ST, light blue: Indoor DT, dark blue: Outdoor DT). Figure 3B shows mean stride time for each participant (*x*‐axis) and condition (light blue: Indoor DT, dark blue: Outdoor DT) individually; error bars indicate the standard deviation across all strides within the respective condition. Figure 3C shows an overlay of normalised stride time across all participants for each stride (light blue: Indoor DT, dark blue: Outdoor DT).

### 2.5 Statistical Analyses

Statistical analyses were conducted using RStudio Version 2024.12.1 + 563 (2009–2025 RStudio, PCB, RRID: SCR_000432). The following R packages were used: *psych*, *stats*, *psycho*, *dplyr*, *devtools*, *rstatix*, *gglpot2*, *ggpubr*, *lsr*, *DescTools*, *svglite*, *tidyr* and *patchwork*. The significance level was set at α = 0.05 for Friedmann tests. Normality of NASA‐TLX scores, d‐prime, response times, speed, stride‐time variability, P2 and P3 amplitudes were assessed using the *Shapiro–Wilk test*. Homogeneity of variances for these measures was evaluated using *F‐tests of variance*. To examine differences across conditions (ST, DT Indoor, DT Outdoor) in NASA‐TLX scores, d‐prime, response times, speed, stride‐time variability, P2 and P3 amplitudes, a Friedman test (for dependent non‐parametric data) was applied. In case the Friedman test indicated significant differences, *Wilcoxon signed‐rank tests* (Wilcoxon 1945) were performed for pairwise comparisons between ST vs. DT Indoor, ST vs. DT Outdoor, and DT Indoor vs. DT Outdoor. Depending on the hypotheses, these tests were used as either two‐tailed or one‐tailed. To control for multiple comparisons, a Bonferroni correction (Bonferroni 1936) was applied, adjusting the significance threshold to α = 0.0125 for Wilcoxon signed‐rank tests. Effect sizes (Cohen's *d* or eta squared) are reported for all significant results.

## 3 Results

### 3.1 Movement

Figure 3B illustrates mean stride time for each participant during the DT Indoor and DT Outdoor conditions, highlighting clear conditions differences in many participants. Figure 3C shows normalised stride time over time, across all participants and strides, separate for the two walking conditions. A systematic time‐on‐task trend in stride time was not visible, neither in DT Indoor nor DT Outdoor.

To assess condition‐related differences in gait performance, walking speed and stride‐time variability were compared between DT Indoor and DT Outdoor conditions. A two‐sided Wilcoxon signed‐rank test showed that walking speed differed significantly (V = 30, *p* < 0.001) between conditions. Descriptively, participants walked faster during DT Outdoor than during DT Indoor. On average across all participants speed was 3.80 ± 0.77 km/h during DT Indoor and 4.37 ± 0.58 km/h during DT Outdoor. Stride‐time and stride‐time variability were additionally assessed to examine differences in gait between DT Indoor and DT Outdoor conditions (see Figure 3). Stride‐time variability did not differ significantly between DT Outdoor and DT Indoor (V = 144, *p* = 0.093). Stride‐time variability was 0.049 ± 0.023 s during DT Indoor and 0.070 ± 0.046 s during DT Outdoor.

### 3.2 Behavioural Results

To test the second hypothesis, Go/No‐Go task performance was assessed by comparing the dependent variables sensitivity (d‐prime) and response times between ST, DT Indoor and DT Outdoor conditions. A Friedman test for dependent data revealed significant effects for both d‐prime (*χ* ^2^(2) = 20.5, *p* < 0.001, η^2^ = 0.706) and response times (*χ* ^2^(2) = 7.7, *p* = 0.021, η^2^ = 0.265). D‐prime during the Go/No‐Go task was further assessed by comparing ST to DT Indoor and DT Outdoor, as well as DT Indoor to DT Outdoor using one‐sided Wilcoxon signed‐rank tests (see Figure 4). D‐prime was significantly higher during ST than during DT Indoor (V = 384, *p* < 0.001, *d* = 0.673), during ST compared to DT Outdoor (V = 444, *p* < 0.001, *d* = 1.339) and during DT Indoor compared to DT Outdoor (V = 362, *p* = 0.003, *d* = 0.516). The mean hit rate was 96.41 ± 4.61% during ST, 95.36 ± 6.81% during DT Indoor, and 92.59 ± 10.43% during DT Outdoor. The mean false alarm rate was 11.93 ± 11.24% during ST, 19.02 ± 14.29% during DT Indoor, and 21.06 ± 14.87% during DT Outdoor. The response bias was *c* = − 0.41 ± 0.41 during ST, *c* = − 0.49 ± 0.31 during DT Indoor and *c* = − 0.42 ± 0.37 during DT Outdoor.

**FIGURE 4:**
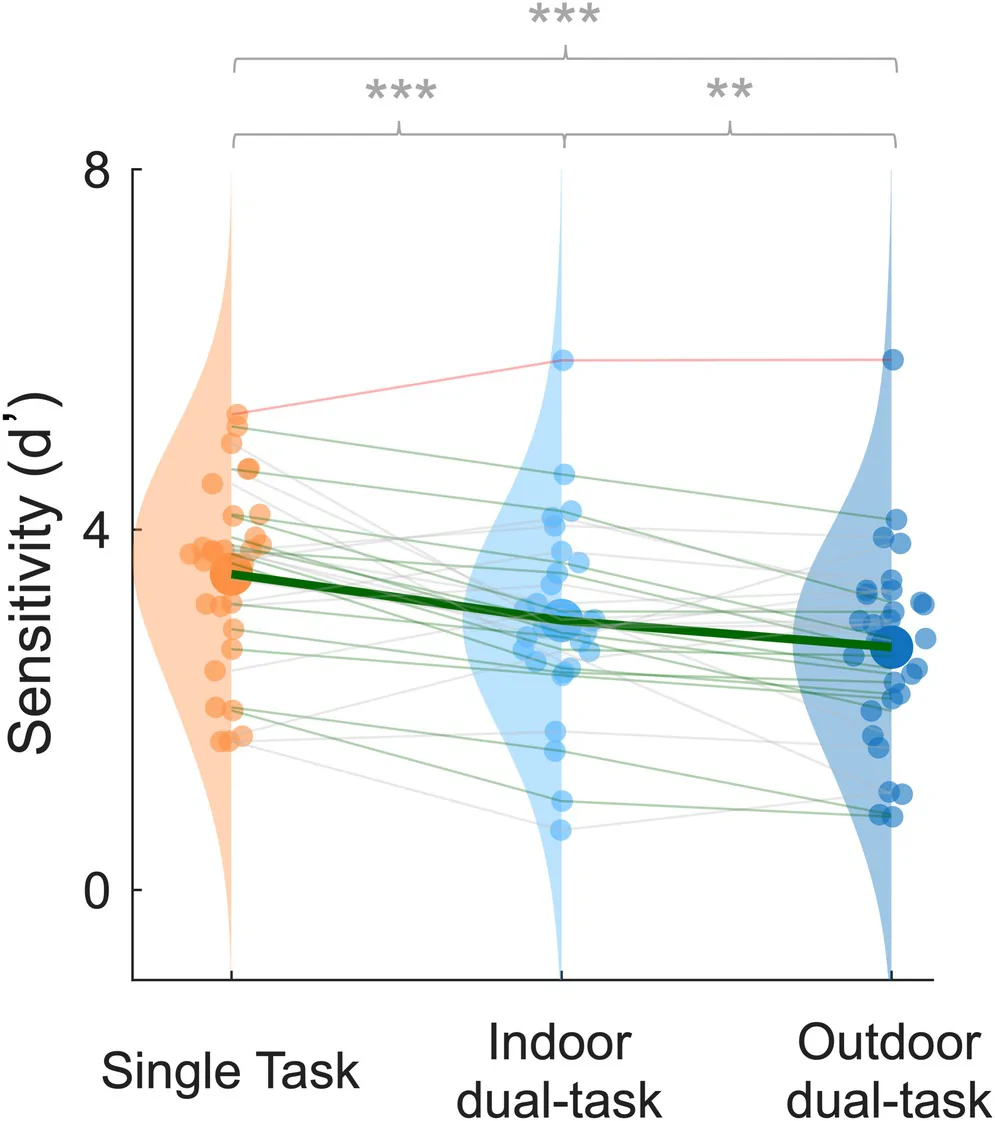
Go/No‐Go sensitivity (d‐prime) distribution across conditions. Small dots represent individual participant mean values during the respective conditions labelled on the *x*‐axis. Connecting lines depict individual trajectories across conditions. Red lines connecting dots mark increased d‐prime from ST to indoor DT to outdoor DT; green lines mark decreased d‐prime from ST to indoor DT to outdoor DT; grey lines mark mixed effects. Larger dots indicate the group mean sensitivity (of individuals means). Significance markers denote *p* < 0.01 (**) and *p* < 0.001 (***).

Response times during the Go/No‐Go task were further assessed by comparing ST to DT Indoor and DT Outdoor, as well as DT Indoor to DT Outdoor (Figure 5). One‐sided Wilcoxon signed‐rank tests were conducted to implement this comparison. While response times were significantly faster during ST than during DT Outdoor (V = 73, *p* < 0.001, *d* = 0.686), they did not differ significantly between ST and DT Indoor (V = 147.5, *p* = 0.162) or DT Indoor and DT Outdoor (V = 134, *p* = 0.036). The median response times (for hits) were 428 ms during ST, 440 ms during DT Indoor, and 476.5 ms during DT Outdoor (data are displayed in Figure 5).

**FIGURE 5:**
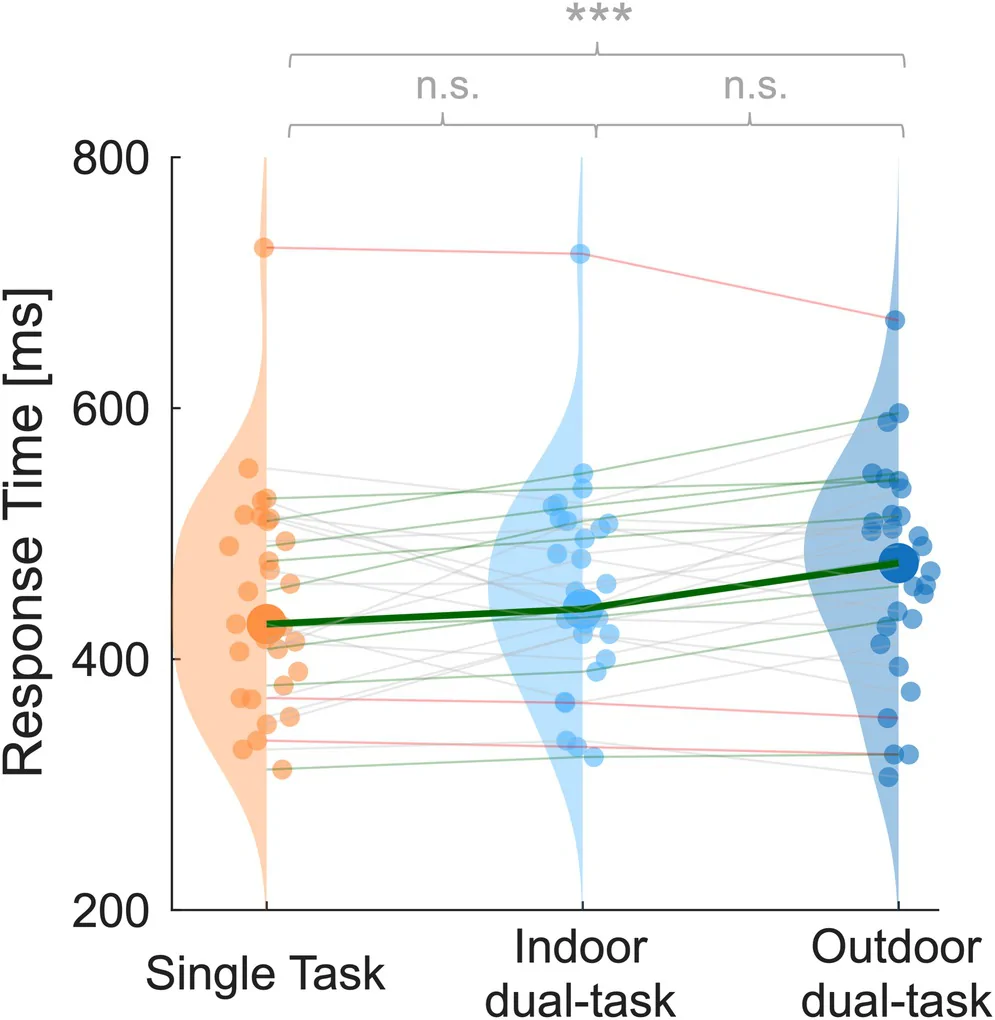
Go/No‐Go response time distribution across conditions. Small dots represent individual participant median values during the respective condition labelled on the *x*‐axis. Connecting lines depict individual trajectories across conditions. Larger dots indicate the group mean response time (of individuals medians). Red lines connecting dots mark decreased response time from ST to indoor DT to outdoor DT, green lines mark increased response time from ST to indoor DT to outdoor DT, grey lines mark mixed effects. n.s. = not significant; *p* < 0.001 (***).

Subjective task difficulty and perceived workload across ST, DT Indoor and DT Outdoor conditions were assessed using the NASA‐TLX questionnaire. Here, participants rated various task‐related demands subjectively (see Figure 6). Mental demand differed significantly between conditions, *χ* ^2^(2) = 22.571, *p* < 0.001 η^2^ = 0.254. Wilcoxon signed‐rank tests showed that mental demand was lower during ST compared to DT Indoor (V = 2.5, *p* < 0.001, *d* = 1.092), while it did neither differ between ST and DT Outdoor (V = 53.5, *p* = 0.018) nor between DT Indoor and DT Outdoor (V = 189.5, *p* = 0.041). Physical demand also differed significantly across conditions, *χ* ^2^(2) = 29.521, *p* < 0.001, η^2^ = 0.332. Wilcoxon signed‐rank tests showed that physical demand was rated significantly lower during ST compared to both DT Indoor (V = 9, *p* < 0.001, *d* = 0.936) and DT Outdoor (V = 4.5, *p* < 0.001, *d* = 0.913). No differences in physical demand were reported between DT Indoor compared to DT Outdoor (V = 158, *p* = 0.141). Temporal demand was also rated significantly different between conditions, *χ* ^2^(2) = 8.881, *p* = 0.012 η^2^ = 0.100. With lower temporal demand during ST compared to DT Outdoor (V = 34.5, *p* = 0.009*, d* = 0.552), but not between ST and DT Indoor (V = 58.5, *p* = 0.047), and DT Indoor and DT Outdoor (V = 83, *p* = 0.639). Effort ratings were different across conditions, *χ* ^2^(2) = 12.628, *p* = 0.002, η^2^ = 0.142, with lower Effort reported for ST compared to DT Indoor (V = 23.5, *p* = 0.001, *d* = 0.652), but no significant differences between ST and DT Outdoor after Bonferroni correction (V = 46, *p* = 0.049) and no differences between DT Indoor and DT Outdoor (V = 132, *p* = 0.315). The Friedmann test indicated that frustration levels varied significantly across conditions, *χ* ^2^(2) = 6.382, *p* = 0.041, η^2^ = 0.072. However, none of the two‐sided post hoc condition comparisons reached significance (ST vs. DT Indoor: V = 65, *p* = 0.138; ST vs. DT Outdoor V = 42, *p* = 0.215; DT Indoor vs. DT Outdoor: V = 74, *p* = 0.181). Performance ratings also differed significantly between conditions, *χ* ^2^(2) = 9.957, *p* = 0.007, η^2^ = 0.112. However, none of the post hoc condition comparisons survived Bonferroni corrections (ST vs. DT Indoor: V = 83.544, *p* = 0.033; ST vs. DT Outdoor: V = 63.5, *p* = 0.119; DT Indoor vs. DT Outdoor: V = 164, *p* = 0.093). Despite these differences, considerable variability was observed across participants in all questionnaire categories.

**FIGURE 6:**
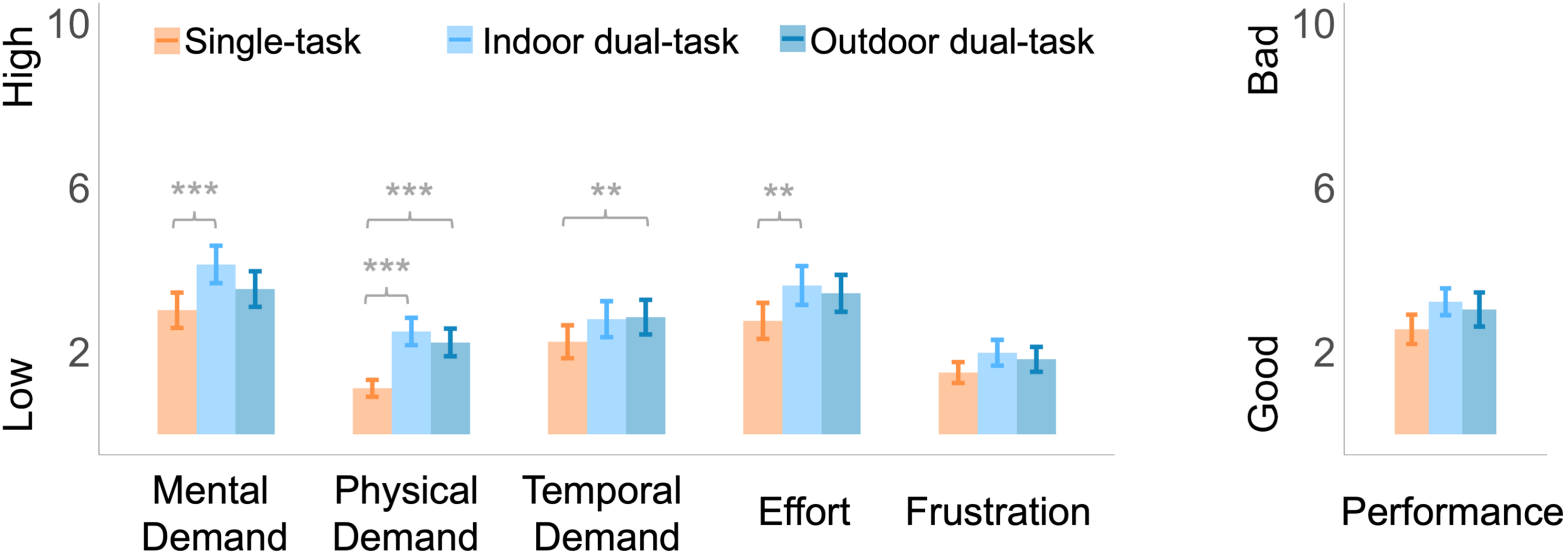
Self‐reported subjective task load (NASA‐TLX) across conditions. Each bar represents the mean subjective task load across participants for each subscale and condition individually. Error bars indicate the standard error of the mean (SEM) across participants. Significance markers denote *p* < 0.01 (**) and *p* < 0.001 (***).

### 3.3 Electroencephalography

As a sanity check, N1 amplitudes were examined to explore whether auditory processing was different between conditions. A Friedman test for dependent data was conducted to compare amplitudes of the N1 auditory evoked potential between ST and DT conditions (Indoor and Outdoor) for both Go stimuli (hit) and No‐Go stimuli (correct rejection) between participants. Neither N1 amplitudes measured for hit trials (*χ* ^2^(2) = 4.967, *p* = 0.088) nor N1 amplitudes for correct rejection trials (*χ* ^2^(2) = 0.8, *p* = 0.670) showed significant differences across the three conditions (see Figures 8 and 10).

To test the third hypothesis on differences in neural processing during the auditory Go/No‐Go task, P2 and P3 amplitudes were compared across ST, DT Indoor, and DT Outdoor conditions (Figures 7, 8, 9, 10). Condition‐related differences in P2 amplitudes following Go stimuli (hits) were further assessed across ST, DT Indoor and DT Outdoor conditions (Figures 7 and 8) using a Friedman test for dependent data. P2 amplitudes (*χ* ^2^(2) = 22.4, *p* < 0.001, η^2^ = 0.772) showed significant differences across the three conditions (see Figures 7 and 8). P2 amplitudes during ST were significantly higher than during DT Outdoor (V = 443, *p* < 0.001, *d* = 1.008), and during DT Indoor than DT Outdoor (V = 426, *p* < 0.001, *d* = 0.852). There were no significant differences between P2 amplitudes recorded during ST and DT Indoor (V = 331, *p* = 0.055). On average across all participants, P2 amplitudes were 4.23 ± 0.44 μV during ST, 3.51 ± 0.42 μV during DT Indoor and 2.24 ± 0.35 μV during DT Outdoor.

**FIGURE 7:**
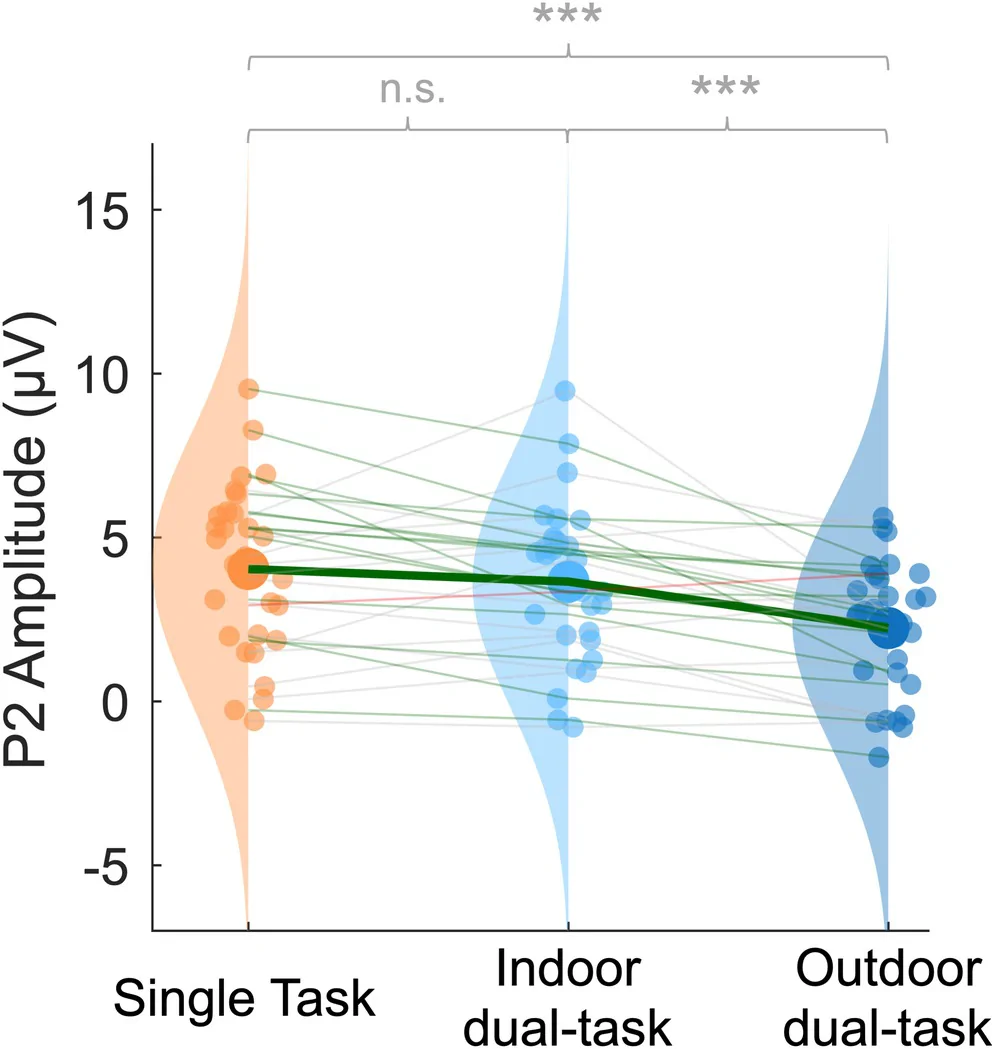
P2 amplitude distribution across conditions. Small dots represent individual participant P2 amplitude during the respective condition labelled on the *x*‐axis. Connecting lines depict individual trajectories across conditions. The larger dot plotted at each condition on the *x*‐axis indicates the mean across participants for that condition. Red lines connecting dots mark increased P2 amplitude from ST to DT Indoor to DT Outdoor; green lines mark decreased P2 amplitude from ST to indoor DT to outdoor DT; grey lines mark mixed effects. Significance markers denote *p* > 0.05 (n.s.) and *p* < 0.001 (***).

**FIGURE 8:**
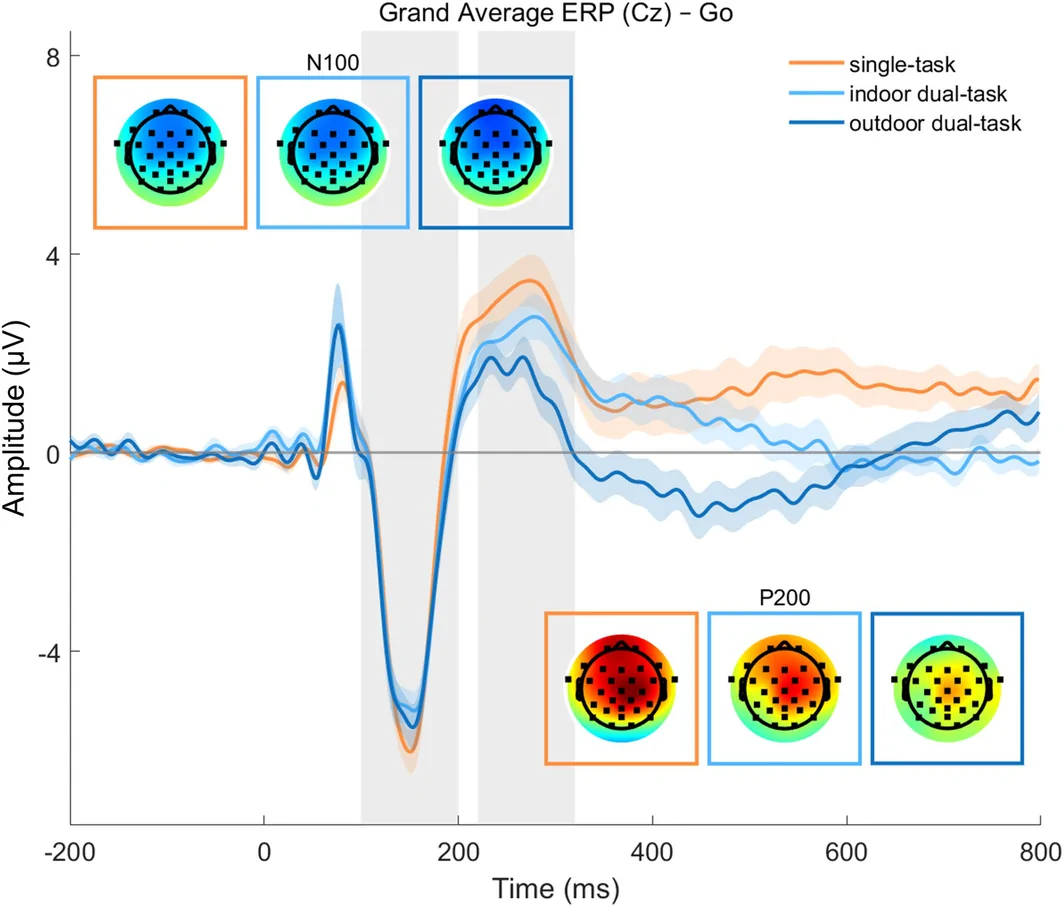
Grand average ERP of all 30 participants at channel Cz for each condition. Grand average ERP while ST (orange; sitting), DT Indoor (light‐blue; treadmill) and DT Outdoor (dark‐blue; overground) are time‐locked to Go stimuli and only include correct responses (hit). The topographies represent an average of the amplitudes of all channels measured between 100 and 200 ms on top (N1, limits −7 to 7 μV) and between 220 and 320 ms on the bottom (P2, limits −2 to 2 μV) either during ST (orange), DT Indoor (light‐blue) or DT Outdoor (dark‐blue). The respective time interval is marked (grey background) in the graphs. N1 and P2 amplitudes are visible in all conditions. The coloured areas surrounding ERPs represent the SEM across participants respectively.

**FIGURE 9:**
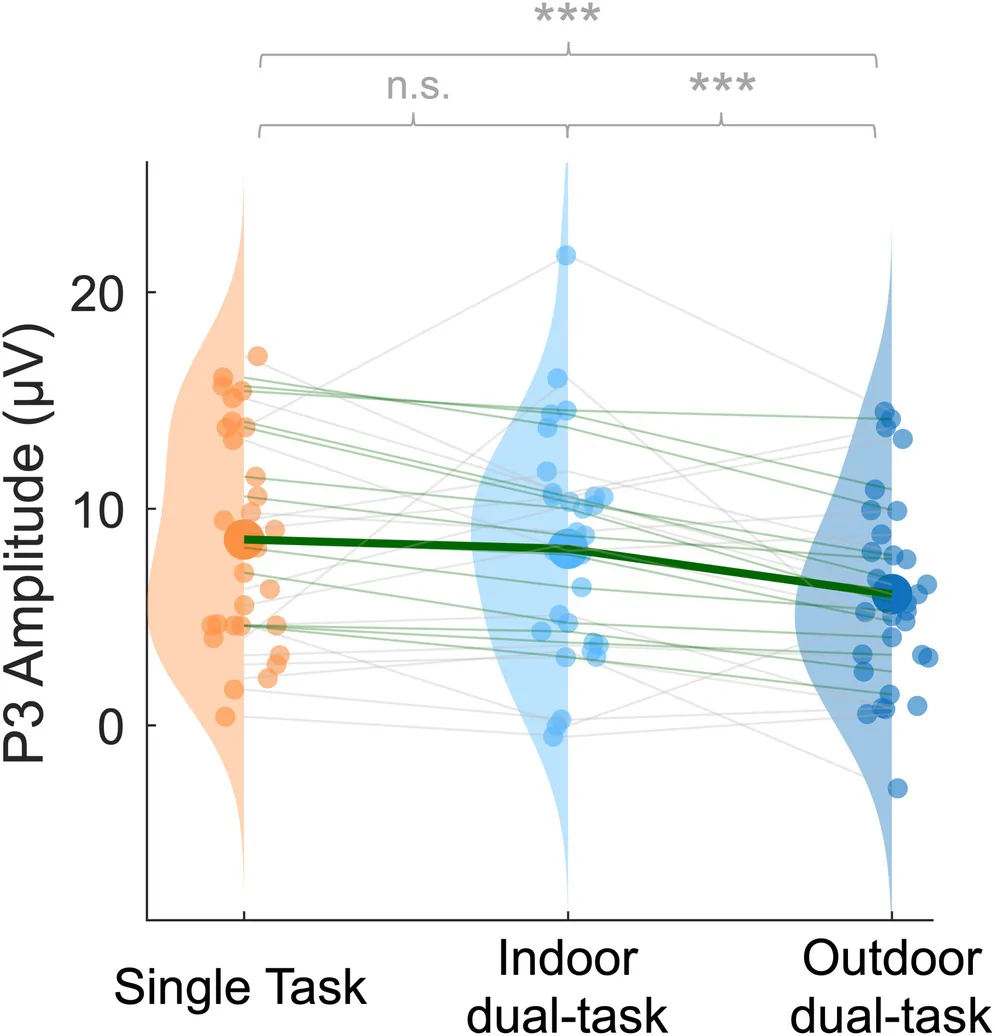
P3 Amplitude distribution across conditions. Small dots represent individual participant P3 amplitude during the respective condition labelled on the *x*‐axis. Connecting lines depict individual trajectories across conditions. The larger dot plotted at each condition on the *x*‐axis indicates the mean across participants for that condition. Green lines connecting dots mark decreased P3 amplitude from ST to DT Indoor to DT Outdoor; grey lines mark mixed effects. Significance markers denote *p* > 0.05 (n.s.) and *p* < 0.001 (***).

**FIGURE 10:**
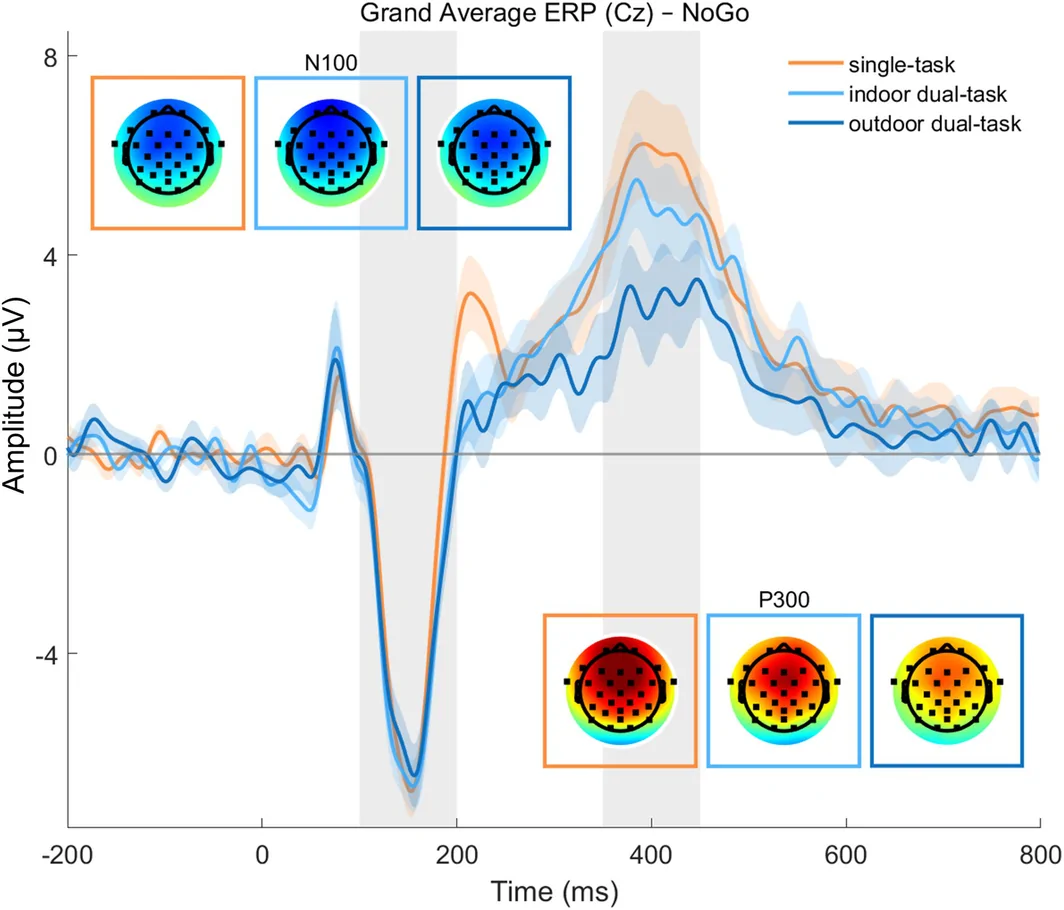
Grand average ERP of all 30 participants at channel Cz for each condition. Grand average ERP while ST (orange; sitting), ST Indoor (light‐blue; treadmill) and DT Outdoor (dark‐blue; overground) are time‐locked to No‐Go stimuli and only include correct responses (correct rejections). The topographies represent an average of the amplitudes of all channels measured between 100 and 200 ms on top (N1, limits −7 to 7 μV) and between 350 and 450 ms on the bottom (P3, limits −7 to 7 μV) either during ST (orange), DT Indoor (light‐blue) and DT Outdoor (dark‐blue), the respective time interval is marked (grey background) in the graphs. N1 and P3 amplitudes are visible in all conditions. The coloured areas surrounding ERPs represent the SEM across participants respectively.

P3 amplitudes following No‐Go stimuli (correct rejections) were also compared across ST, DT Indoor and DT Outdoor conditions (Figures 9 and 10). A Friedman test for dependent data indicated that P3 amplitudes showed significant differences across the three conditions (*χ* ^2^(2) = 20.867, *p* < 0.001, η^2^ = 0.720). With significantly higher P3 amplitudes during ST than DT Outdoor (V = 448, *p* < 0.001, *d* = 1.151), and during DT Indoor than DT Outdoor (V = 384, *p* < 0.001, *d* = 0.613), but no significant differences between P3 amplitudes recorded during ST and DT Indoor (V = 309, *p* = 0.060; see Figures 9 and 10). On average, across all participants, P3 amplitudes were 7.67 ± 0.83 μV during ST, 7.08 ± 0.79 μV during DT Indoor and 4.84 ± 0.71 μV during DT Outdoor.

## 4 Discussion

The present study investigated how motor and environmental modality influence cognitive–motor interactions in older adults walking on a treadmill and outdoors. When compared to a stationary sitting condition, the findings provide partial support for the hypothesis that DT demands impair cognitive performance. Interestingly, stronger DT impairments were found in the more naturalistic outdoor condition.

In line with our hypothesis, d‐prime decreased from ST to DT Indoor to DT Outdoor, suggesting reduced sensitivity under increasing task demands. A bias analysis revealed that response bias (c) was below zero across all conditions, indicating a general tendency toward a liberal yes‐saying response strategy. Participants were thus more likely to respond to tones even under uncertainty, which may reflect a prioritisation of speed over accuracy in the auditory Go/No‐Go task. Response times descriptively followed the expected pattern, increasing across conditions from ST to DT Indoor to DT Outdoor. However, apart from significantly faster responses in ST compared to DT Outdoor, differences between ST and DT Indoor as well as between DT Outdoor and DT Indoor did not reach significance or did not survive correction for multiple comparisons. This pattern may reflect substantial inter‐individual variability, as several participants showed either the opposite or an altered response time pattern than expected, indicating heterogeneous task strategies. In line with this argument, a similar pattern of variability and context‐dependent strategy shifts has previously been reported in younger adults (Papin et al. 2026). It may well be that the DT Indoor condition did not require more overlapping processing demands with the cognitive task than the ST condition, resulting in differences that were too small to be detected with the given sample size. Further, the DT Outdoor condition likely introduced additional variability due to environmental factors such as distractions and visual flow, leading to more inconsistent response times across the trials and making differences between DT conditions harder to detect. This interpretation aligns with findings by Liebherr and colleagues, who demonstrated that increasing environmental complexity modulates attentional processing, even when behavioural differences remain subtle (Liebherr et al. 2021).

At the neurophysiological level, P2 and P3 amplitudes descriptively decreased from ST to DT Indoor to DT Outdoor, consistent with the assumption of CMI (Kok 2001; Polich 2007). However, significant differences were primarily observed between ST and DT Outdoor, and between DT Indoor and DT Outdoor, whereas differences between ST and DT Indoor did not reach statistical significance. The pattern of results may point toward differences in environmental and motor demands between conditions influencing DT outcomes. Treadmill walking, due to its externally paced and constrained nature, likely reduces the need for adaptive control processes. In contrast, overground walking requires continuous adjustment to environmental conditions, including navigation and processing of visual flow (Lang et al. 2026). This is in line with the scoring guidelines Cinar and colleagues introduced where overground walking would be rated as requiring higher demands in visuospatial perception, visuospatial cognition, the vestibular domain and locomotor response when compared to the specific treadmill used in this study (Cinar et al. 2022). The reduction of these demands in treadmill walking may explain the reduced differentiation (i.e., descriptive but not significant) between ST and DT Indoor conditions. In addition, self‐paced walking has been shown to increase CMI effects, possibly due to the attentional cost associated with regulating one's own pace (S.‐C. Kao et al. 2020), which may further contribute to the stronger effects observed in the DT Outdoor condition.

Importantly, gait speed differed significantly between conditions. Therefore, these findings could also be explained by previous mobile EEG research suggesting that gait speed itself influences neurophysiological measures, such as ERP amplitudes and spectral power, during locomotion (Lee et al. 2021; Nordin et al. 2019). For example, Nordin et al. (2019) reported reduced auditory electrocortical responses, including N1 and P3 amplitudes, with increasing treadmill walking speed, suggesting that gait speed may affect the processing of auditory information during walking. In the present study gait speed was faster during DT Outdoor compared to DT Indoor. Hence, we cannot exclude the possibility that differences in gait speed contributed to the observed behavioural and neurophysiological effects. However, in contrast to the findings of Nordin et al. (2019), the present study did not provide evidence for a general effect across all ERP components, as in the current study N1amplitude did not differ between conditions. Thus, although the reduction in P3 amplitude observed during indoor walking could potentially be related to the slower walking speed, the absence of a corresponding N1 effect suggests that the influence of walking speed may not extend uniformly across ERP components. Moreover, previous work by Kline et al. (2014) found that neither task performance nor task‐related spectral power during a spatial working memory task was significantly affected by walking speed across a range of walking speeds (Kline et al. 2014), suggesting that differences in gait speed do not necessarily alter cognitive task performance or its associated neural activity. Furthermore, subjective workload ratings provide important context for interpreting this potential confounding factor. Based on NASA‐TLX measures, participants rated physical demand lower during ST compared to both DT conditions, but ratings did not differentiate between DT Indoor and DT Outdoor, despite the higher walking speed during DT Outdoor. We assume that, although gait speed differed objectively, participants did not perceive the DT Outdoor condition as more physically demanding than DT Indoor, indicating that subjective task demands were comparable across DT conditions. Unfortunately, indoor and outdoor single motor task conditions were not included due to time constraints. Contrasting DT and ST gait speed would reveal further insights on how well task demands are captured by gait speed alterations.

The behavioural and neurophysiological findings taken together confirm that DT costs are present, but depend on environmental context. In the present study, both the walking tasks and the concurrent auditory Go/No‐Go likely recruited overlapping cognitive resources, suggesting a shared resource pool and therefore a basis for CMI. While both DT conditions involve concurrent cognitive and motor demands, the DT Outdoor condition appears to involve greater overlap between the resources recruited for locomotion and those required by the cognitive task, leading to stronger CMI effects, visible in both behaviour and ERPs. This interpretation is consistent with previous studies showing reduced ERP amplitudes during DT walking outdoors, compared to sitting (De Vos et al. 2014; Debener et al. 2012; Reiser et al. 2019). Additionally, Reiser et al. (2021) showed that increased motor demands reduce cognitive performance under DT conditions. This observation also aligns with a study done by Liebherr et al. (2021), who reported progressively reduced P3 amplitudes from controlled laboratory to real‐world environments. Notably, their findings also showed that behavioural performance can remain relatively stable despite substantial neurophysiological changes, highlighting the importance of considering both levels of analysis. Here we could show changes in both behaviour (d‐prime) and neurophysiology which may highlight differences between older adults and younger adults studied by both Reiser et al. (2021) and Liebherr et al. (2021). Rather than just simply reflecting increased cognitive load, differences between indoor and outdoor DT may also point to qualitative shifts in attentional allocation and task strategy across contexts (Papin et al. 2026). Although treadmill walking has been shown to elicit greater activation of prefrontal and motor areas than overground walking, suggesting that it may involve different and potentially more demanding neuromotor control processes (Herold et al. 2019), this does not seem to necessarily result in greater DT effects. From a theoretical perspective, Wickens' MRT proposes that interference arises when concurrent tasks compete for shared resources across processing stages, modalities and processing codes (Wickens 2008, 2024). Thus, these greater overall task demands reported by Herold et al. (2019) do not necessarily lead to greater DT effects; rather, interference is expected primarily when the resources required by the concurrent tasks overlap. From this perspective, treadmill and outdoor walking may recruit different types of resources, with the additional visuospatial processing, environmental monitoring, and continuous adaptation required during outdoor walking potentially resulting in greater overlap with the resources required for the secondary cognitive task.

Contrary to our hypothesis, stride‐time variability did not differ significantly between conditions, while participants walked faster during DT Outdoor compared to DT Indoor. The higher gait speed observed during DT Outdoor is consistent with previous findings (Schmitt et al. 2021), suggesting that overground walking is generally less constrained and allows for more natural locomotor behaviour than treadmill walking. Rather than reflecting increased variability, the comparable stride‐time variability across conditions indicates that participants maintained a similar level of temporal gait stability despite the higher DT Outdoor speed. This pattern is consistent with the idea that treadmill and overground walking impose fundamentally different locomotor constraints (Carpinella et al. 2010; Chai et al. 2023; Hollman et al. 2016), which may influence resource allocation and the magnitude of motor demands under DT conditions. However, without a ST walking condition, it remains difficult to determine to what extent the addition of the cognitive task differentially affected gait parameters across environments. Future studies should therefore include ST walk conditions to better isolate ST interference effects on gait.

The present study supports the notion that treadmill walking does not fully capture real‐world DT behaviour, potentially leading to an underestimation of DT costs in everyday life. While treadmill paradigms offer high experimental control, they may inadequately reflect the complexity and introduce different demands than everyday walking, which involves continuous adaptation to dynamic and unpredictable environments. This observation is consistent with studies showing differences in gait variability and control between treadmill and overground walking (Carpinella et al. 2010; Hollman et al. 2016; Lazzarini and Kataras 2016). Moreover, the framework proposed by Cinar and colleagues, in which the application of the scoring criteria results in higher demand scores for overground than for treadmill walking and thereby expecting higher DT costs during overground walking further supports this notion (Cinar et al. 2022).

The scoring guidelines mentioned above may also help explain variability and seemingly contradictory findings across studies. By explicitly accounting for factors such as walking‐path width, the guidelines acknowledge that different locomotion setups can impose different task demands on participants, which may already differ depending on the specific treadmill used in an experimental setup. Furthermore, treadmills and overground walking paths may also differ in width and environmental constraints, thereby altering the degree of cognitive‐ and motor control required for walking. More generally, variations in motor task demands (e.g., treadmill vs. overground walking) and cognitive task characteristics (e.g., auditory vs. visual processing) may influence the degree of task conflict and, consequently, the magnitude and direction of cognitive–motor interactions. Such methodological differences may therefore contribute to discrepancies in reported outcomes across studies. This may help explain why some studies report facilitation effects (Patelaki, Foxe, Mantel, et al.^2023^; Patelaki, Foxe, Mazurek, and Freedman 2023), whereas others, including the present study, observe CMI. Considering these factors may improve the comparability of future studies and facilitate a more nuanced interpretation of cognitive–motor interaction effects across different experimental contexts.

Taken together, the findings of the present study highlight the importance of systematically varying one task component when investigating cognitive–motor effects. Our approach of comparing treadmill and overground walking, while keeping cognitive task demands constant, helped isolate the specific contribution of walking modality to DT performance. Future research should increasingly leverage portable, unobtrusive technologies (Bleichner and Debener 2017), such as mobile EEG and IMUs, to assess DT behaviour in everyday life.

## 5 Limitations

A major limitation of the present study is that walking speed was not equivalently controlled across conditions. While treadmill walking was performed at a fixed, externally imposed pace due to the electronic control of the device, participants walked at a self‐selected speed during the DT Outdoor condition. Although they were instructed to match their treadmill pace, most participants preferred to walk faster outdoors. This mismatch reduces the comparability of gait parameters between conditions, as speed differences may have influenced both motor and cognitive outcomes. Furthermore, differences in gait speed may have contributed to the observed differences in ERP amplitudes, as previous research has shown that walking speed may modulate ERP amplitudes (Lee et al. 2021; Nordin et al. 2019). Moreover, the fixed treadmill speed does not allow for natural, dynamic adjustments in pace that typically occur during overground walking, further emphasising the differing locomotor constraints between conditions.

In addition, the absence of a ST walking condition limits the interpretation of DT effects on gait. Consequently, the accelerometer‐derived measures can only be interpreted as descriptive or control parameters rather than as direct indicators of DT interference. Future studies should therefore include all single‐ and dual‐task conditions across cognitive and motor domains to more clearly disentangle the specific effects of dual‐tasking on gait and cognition.

## 6 Conclusion and Outlook

Future studies should further disentangle the contributions of motor and environmental demands by including both treadmill and ecologically valid overground conditions, as well as ST walking baselines. Importantly, the potential influence of gait speed on neurophysiological measures also warrants further investigation, as differences in walking speed between conditions may contribute to ERP amplitude differences. Nevertheless, the present findings suggest that treadmill and overground walking are not interchangeable; they differ in key aspects such as visual flow, environmental variability and adaptive control requirements, and these factors can in turn influence dual‐task performance.

In healthy aging, structural and functional changes reduce the automaticity of motor control (Beurskens and Bock 2012; Rosso et al. 2025). As a result, gait increasingly relies on prefrontal, top‐down control processes. Consistent with this shift, Kahya et al. (2019) report increased prefrontal activation during DT gait in older adults and clinical populations, commonly interpreted as compensatory cognitive recruitment (Kahya et al. 2019), which is associated with increased CMI and risk of falls (Bayot et al. 2020). This vulnerability may be further amplified in Parkinson's disease, where degeneration of dopaminergic pathways disrupts both motor and cognitive processes (Witt et al. 2006). Consequently, individuals with Parkinson's disease often exhibit pronounced gait impairments and an elevated fall risk, particularly under DT conditions (Kahya et al. 2019; Kelly et al. 2012). DT paradigms such as those employed here may therefore represent a valuable tool for geriatric and neurological assessment, as DT gait performance has been shown to correlate with cognitive decline (Ramírez and Gutiérrez 2021) and may help inform targeted interventions.

Despite the clear relevance of DT behaviour for everyday functioning, much of the existing research relies on treadmill‐based paradigms (Chai et al. 2023; P.‐C. Kao and Pierro 2021), which may underestimate real‐world cognitive–motor demands due to their constrained and less variable nature. This is supported by the present findings, which show stronger effects during natural overground compared to treadmill walking, indicating that environmental context and walking modality substantially modulates CMI. Accordingly, laboratory‐based assessments do not fully capture the complexity of real‐world walking. Future research should therefore prioritise more ecologically valid, real‐world approaches using mobile and wearable technologies such as smartphone‐based sensing, IMUs, and mobile EEG. These methods enable the assessment of cognitive–motor behaviour in everyday life. We hope that they can contribute to the development of sensitive diagnostic tools and targeted interventions for cognitive decline and neurodegenerative diseases.

## Author Contributions

**Lara J. Papin:** conceptualization, writing – original draft, methodology, validation, visualization, writing – review and editing, formal analysis, project administration, data curation. **Julius Welzel:** conceptualization, writing – review and editing, validation, supervision, methodology. **Jessica Koschate‐Storm:** conceptualization, writing – review and editing, validation, methodology, funding acquisition. **Tania Zieschang:** conceptualization, methodology, validation, writing – review and editing, funding acquisition. **Karsten Witt:** conceptualization, writing – review and editing, validation, methodology, supervision, funding acquisition. **Stefan Debener:** supervision, funding acquisition, conceptualization, validation, methodology, writing – review and editing, project administration.

## Funding

This project was supported by the Deutsche Forschungsgemeinschaft (DFG, German Research Foundation). LJP, material and participant honorarium were funded by the Research Training Group (RTG) 2783, Project ID 456732630. SD was funded under Germany's Excellence Strategy – EXC 2177/1, Project ID 390895286. The M‐Gait treadmill (Motek) was funded by the Federal Ministry of Education and Research (BMBF), Grant Number: 01GY2021.

## Conflicts of Interest

The authors declare no conflicts of interest.

## Supporting information


**Figure S1:** Outdoor walking route on the university campus. Participants walked alongside a large campus building on a uniformly paved, even surface throughout the route. The two smaller images on the left and right provide representative views of the walking surface.

## Data Availability

Data will be made available on request.
