# Supplementary material for: Treadmill or Overground? Cognitive–Motor Interference Effects in Healthy Older Individuals
Source: Eur J Neurosci. 2026 Oct 25;64(8):e70724. doi: 10.1111/ejn.70724 (PMC13696155; doi:10.1111/ejn.70724)
Supplement: Supplementary file 1 [file EJN-64-0-s001.docx]

**Supplementary Material**

Supplementary Figure 1: Outdoor walking route on the university campus. Participants walked alongside a large campus building on a uniformly paved, even surface throughout the route. The two smaller images on the left and right provide representative views of the walking surface.
